# Contact-free determination of viscosity in multiple parallel samples

**DOI:** 10.1038/s41598-019-44859-z

**Published:** 2019-06-06

**Authors:** Michaela Sieben, René Hanke, Jochen Büchs

**Affiliations:** RWTH Aachen University, AVT – Biochemical Engineering, Aachen, Germany

**Keywords:** Chemical engineering, Characterization and analytical techniques, Applied physics

## Abstract

Viscosity is an inherent characteristic of fluids and is therefore an important parameter in many different processes. Current methods to measure viscosity involve direct contact with the liquid sample, which is often undesirable. Here we present a simple, precise and robust contact-free method to determine viscosity, using a single drive motor, inexpensive components and disposable sample vessels. The measurement principle involves the detection of viscosity-dependent angular positions in a rotating liquid relative to the direction of centrifugal acceleration in an orbitally shaken vessel. The signal can be detected using different optical methods, as shown here using fluorescence and transmitted light. The sensitivity of the system can be adjusted over a wide range by varying the sample volume or the shaking diameter, and multiple samples can be analysed in parallel. This novel viscometer is also applicable to characterize non-Newtonian shear rate-dependent fluids.

## Introduction

The rheological behaviour of viscous liquids is relevant in the food^1–4^, pharmaceutical^5–9^ and cosmetic^10–12^ industries, as well as technical chemistry^13–15^ and production engineering^16^. Several devices have been developed to measure the viscous, viscoelastic and elastic properties of liquids and solids^17–19^. The most common are pressure-driven and drag flow viscometers^16^. Typical pressure-driven viscometers include flow cups, falling sphere viscometers and glass capillary viscometers. In all three cases, flow is induced by gravitational force and measurement is dependent on weight and density, and can only be determined for Newtonian fluids with low viscosity^3^. Falling sphere viscometers additionally require transparent samples, unless variants based on induction or electromagnetic fields are available^19–21^. Drag flow viscometers include spindles and mixer-type rheometers. Spindles are immersed in the sample and rotated at a specified rate to measure the resulting torque^19^. Different geometries are available (cylinders, disks, cones, pins and T-bars) and such devices are often used for quality assurance^22–24^. Mixer-type rheometers with various rotating stirrers are particularly suitable for the analysis of building materials and foodstuffs containing large dispersed particles, e.g. fruit pieces or sand^25,26^.

Viscometers can also be classed as absolute or relative measuring systems. The former have a standard “two plates” geometry and defined shear conditions in a relatively narrow shearing gap, whereas the latter do not meet the conditions of the two-plates model^17^. The calculation of parameters such as shear stress, shear rate and viscosity is not recommended in relative measuring systems, but raw data such as torque or speed can be used instead^19^. All the pressure-driven and drag flow viscometers described above are relative measuring systems. Absolute measuring systems include rotation and oscillation rheometers that use concentric cylinders, plate-plate or cone-plate systems to determine the flow behaviour of Newtonian and non-Newtonian fluids. Concentric cylinders consist of a measuring cup and a bob with a narrow gap, and can be defined as Couette type (outer part rotates) or Searly type (inner part rotates)^18^. Typically, concentric cylinders are used for low-viscosity or particulate systems and are difficult to clean^19,27^. The plate-plate system consists of fixed and rotating plates with an adjustable gap and can be used with particulate and high-viscosity systems^17^. Instead of a rotating plate, cone-plate systems use a truncated cone as a rotating element, resulting in a conical gap and uniform shearing of the sample over the conic radius^19^. Typically, cones with a radius of 10–30 mm are used with a 0.3–6° gap angle^28^. The gap height in the centre depends on the angle and the radius, and is in the range 50–200 µm^17^. Cone-plate systems are versatile but they are unsuitable for fluids containing dispersed particles larger than the gap size^17^. These advanced rotation and oscillation rheometers are more expensive and more sensitive than the simpler pressure-driven and drag flow viscometers because they contain components such as air bearings. They also have limitations such as inaccurate measurement of the true sample radius^18,29^, hydrodynamic instability^30,31^, low torque limit^32,33^ and wall slip behaviour^34^.

## Results

### Liquid distribution as a function of viscosity

**Figure 1:**
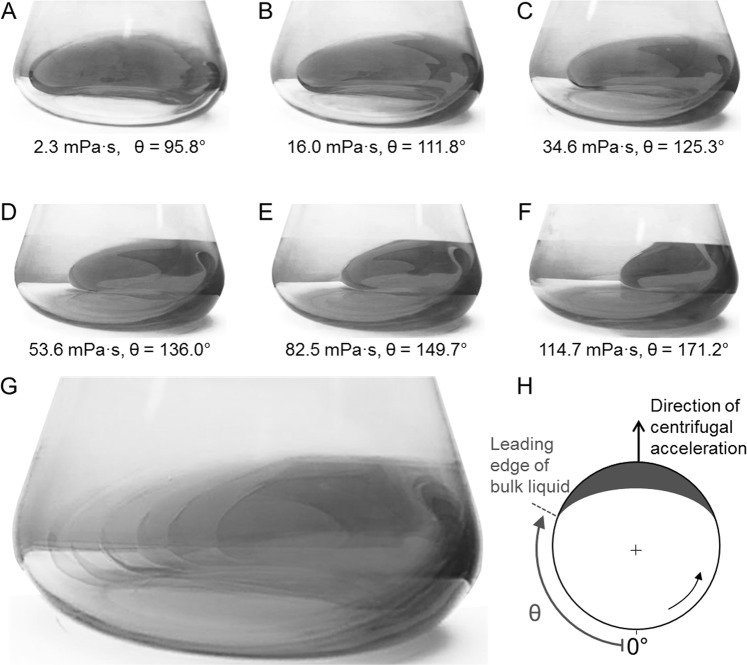
(**A**–**F**) Liquid distribution in a rotating shake flask at increasing viscosities. PVP solutions of different concentrations (Supplementary Information Table S1), flask volume = 250 mL, liquid volume = 30 mL, shaking frequency = 150 rpm, shaking diameter = 50 mm, temperature = 25 °C. Photographs were taken in the direction of the centrifugal acceleration using a rotating camera^48,49^. The corresponding viscosity is indicated below each panel together with the angle θ (defined in H). (**G**) Superimposition of the images in panels (A–F) to visualize the increasing angle offset of the bulk liquid in relation to the direction of centrifugal acceleration for increasing viscosities. (**H**) Schematic top view of a shake flask with rotating liquid, where θ indicates the angle between the zero point and the leading edge of the rotating bulk liquid. The shake flask was placed in an orbital shaker with a counter-clockwise rotational path. Figure adapted from Sieben^19^.

Here we describe a new method for the quantitative determination of viscosity (η) which addresses the limitations of current viscometers. The measurement principle is based on detecting the angular position of a liquid sample in an orbitally shaken vessel relative to the direction of centrifugal acceleration. Although other geometries (such as cylinders) are suitable, we chose Erlenmeyer flasks as our standard sample vessels because they are easy to handle and well characterized^35–39^. The translational orbital movement of the sample vessel on a circular path causes the bulk liquid of the sample to rotate due to its inertia^19^. Simultaneously, a thin liquid film adheres to the inner flask wall, if the liquid is aqueous and the wall is a hydrophilic material such as glass^39–41^. At water-like viscosities, the bulk liquid of the sample rotates in phase with the shaker, its centre of mass pointing in the direction of centrifugal acceleration (Fig. 1A,H). With increasing viscosity (Fig. 1B–F), the hydrodynamics in the sample vessel change due to the greater friction between the liquid and the glass wall of the vessel, causing a phase-shift in the bulk liquid relative to the direction of centrifugal acceleration. High viscosity can trigger the complete collapse of liquid movement and the system turns “out-of-phase^19,36^”. This flow regime is unsuitable for viscosity measurement. The critical viscosity at which the out-of-phase condition appears depends on the shaking conditions. The phase number^36^ distinguishes between in-phase (phase number > 1.26) and out-of-phase (phase number < 1.26) operating conditions, and thus determines the critical viscosity.

Figure 1G shows an overlap of Fig. 1A–F revealing how the rotating bulk liquid shifts with increasing viscosity relative to the direction of the centrifugal acceleration. The position of the leading edge of the bulk liquid correlates directly with the viscosity of a Newtonian liquid and forms the basis of the measuring device described herein^19^. For non-Newtonian liquids, we can assess the apparent (shear rate-dependent) viscosity. Because the leading edge of the bulk liquid has a distinct geometry, it is easily detected and taken as a reference point for the detection of the liquid’s angular position. The angle between the zero point and the leading edge of the rotating bulk liquid is defined as θ (Fig. 1H)^19^.

### Viscosity measurement device

**Figure 2:**
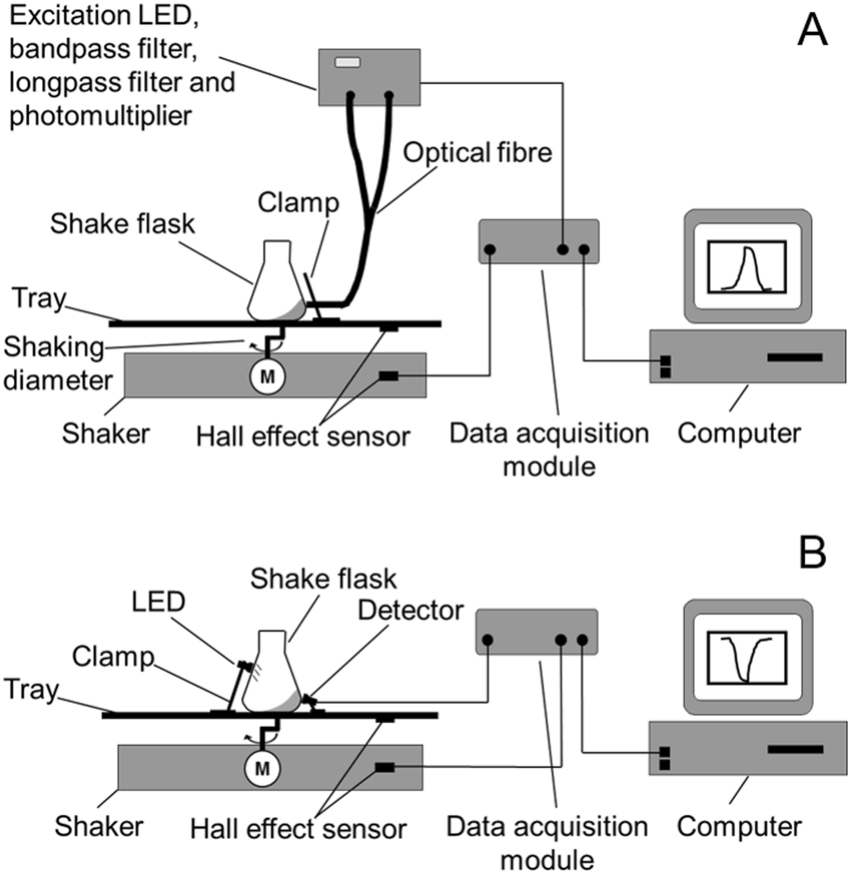
Schematic representation of the measurement setup for fluorescence and transmitted light. In both cases, the shaker housing was equipped with a self-built Hall effect sensor on the longitudinal axis and a magnet on the rotating eccentric disk. When the magnet on the eccentric disk passed the Hall effect sensor, the signal dropped. The combination of Hall effect sensor and magnet served as a counter and position marker. (**A**) Fluorescence measurement. Incident light (excitation wavelength) and fluorescence (emission wavelength) passed through the same optical glass fibre system. (**B**) Transmitted light measurement. The light source and detector are positioned on opposite sides of the shake flask. Figure adapted from Sieben^19^.

The angular position of the bulk liquid’s leading edge can be detected using various contact-free optical methods, and herein we describe one method based on fluorescence (Fig. 2A) and another based on transmitted light (Fig. 2B). In both cases, the sample vessel (a 250-mL Erlenmeyer flask without graduations) was mounted on a LS-X orbital shaker (Kühner AG, Birsfelden, Switzerland) equipped with a Hall effect sensor as an angular position marker. The shaker was placed in a temperature-controlled hood. Fluorescence measurement was achieved by supplementing the liquid with a fluorescent dye, e.g. fluorescein (*λ*_*ex*_ = 490 nm, *λ*_*em*_ = 514 nm^42^). Light from an LL-504BC2E-B4-2CC blue high-power LED (*λ*_*max*_ = 470 nm; LuckyLight Electronics, Co., Ltd, Shenzhen, China) was passed through a BG 12 band-pass filter (*λ*_*max*_ = 470 nm; Schott AG, Mainz, Germany) and via an optical fibre to the glass wall of the shake flask to excite the fluorescent dye^19^. The optical fibre was mounted 26.6 mm from the base of the shake flask and oriented perpendicularly to the sloping shake flask wall. The excited fluorescent dye emits yellow light that feeds back via the optical fibre through a OG 515 long-pass filter (*λ* > 515 nm, Schott AG) to a custom-made photomultiplier (PreSens Precision Sensing GmbH, Regensburg, Germany). The photomultiplier signal was amplified (gain = 350) and converted into a voltage signal. This signal along with the position signal from the Hall effect sensor was forwarded via a PC Scope 500 data acquisition module (Vellemann nv, Gavere, Belgium) to a computer with a PC Lab 2000 SE graphical interface (Vellemann nv) to be plotted and stored^19^.

Whereas the fluorescence measurement requires the sample to be mixed with a fluorescent dye, the transmitted light measurement is generally applicable for all aqueous samples (Fig. 2B). Based on the principle of a light barrier, the light source and detector were placed on opposite sides of the shake flask. Any liquid moving between the light source and detector reduces the amount of light that reaches the detector due to optical absorption. We used an LD 274 near-infrared LED (*λ*_*max*_ = 950 nm; OSRAM Licht AG, Munich, Germany) to take advantage of the liquid water absorption band at ~970 nm^19,43–45^. The LED was mounted 70 mm above the maximum liquid height to avoid any interference from the liquid on the illumination side of the shake flask. The corresponding 203 FA infrared detector (OSRAM Licht AG) was fixed 13 mm from the base of the shake flask on the side opposite the LED. This height represents the maximum radial extension of the flask as its truncated cone merges into the quarter torus. The liquid reaches its maximum circumferential extension within the shake flask at this height (Fig. 1) making this position ideal for measurement. From the detector, the signal was forwarded together with the Hall effect sensor signal to an NI USB 6210 data acquisition module (National Instruments, Austin, TX, USA) that transmitted the data to a computer running LabVIEW™-based software (National Instruments) to visualize and store the data^19^.

The leading edge of the rotating bulk liquid can also be detected by a radar sensor (data not shown) which is compatible with non-transparent sample vessels.

### Optical signals and measurement of the angle θ

**Figure 3:**
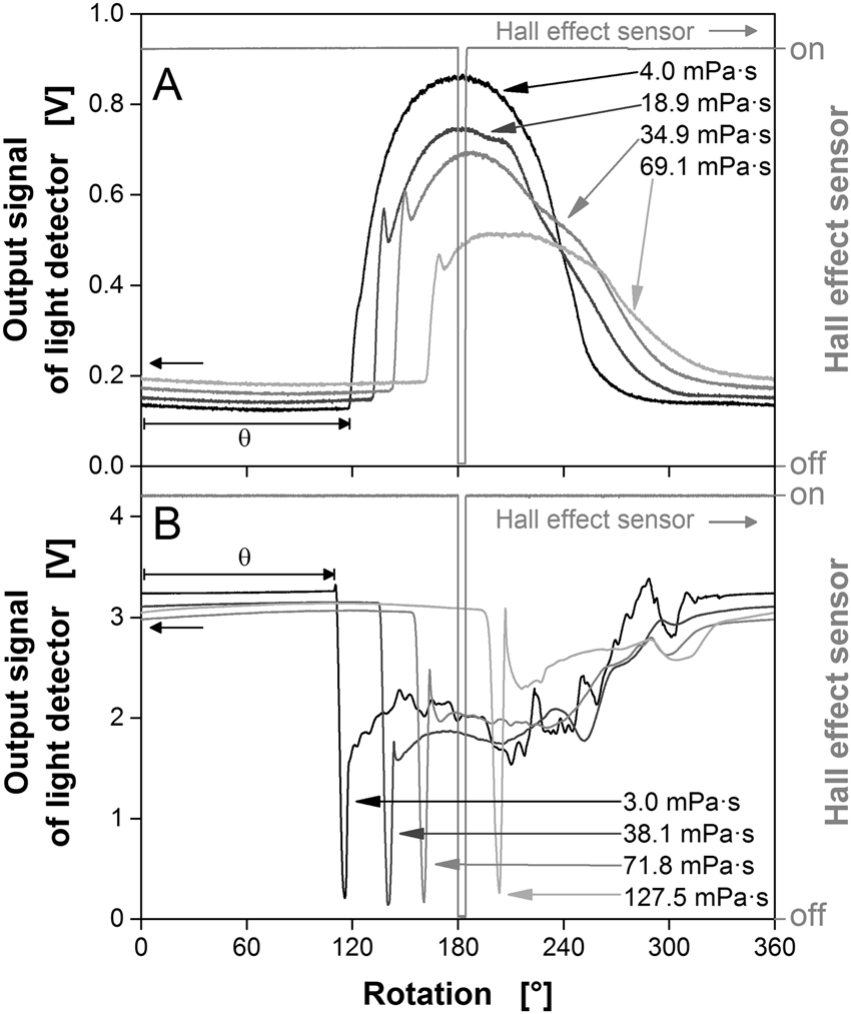
Raw signals measuring the liquid distribution in shake flasks. PVP solutions of different concentrations (Supplementary Information Table S2, Supplementary Information Table S3), flask volume = 250 mL, liquid volume = 20 mL, shaking frequency = 350 rpm, shaking diameter = 50 mm. (**A**) Fluorescence measurement (Fig. 2A), temperature = 37 °C, 2.5 µM fluorescein, 100 mM phosphate buffer (pH 8.0), optical fibre 26.6 mm from the flask base; (**B**) Transmitted light measurement (Fig. 2B), temperature = 30 °C, detector 13 mm from the flask base. Figure adapted from Sieben^19^.

Figure 3 shows optical signals representing the liquid distribution for the fluorescence and transmitted light measurements in shake flasks. In each case, the raw signal (output signal from the light detector) is a function of the angle of four aqueous polyvinylpyrrolidone (PVP) solutions of different concentrations and viscosities during one rotation. The signal of the Hall effect sensor is shown as a position marker for the orbital shaker. The left flank of the signal indicates the direction of centrifugal acceleration. The origin of the angular position is defined as indicated in Fig. 1H.

The signals obtained using both measurement principles were initially constant and formed a horizontal line. Under these conditions, no bulk liquid crosses in front of the optical fibre or the infrared detector, with the exception of a thin liquid film adhering to the flask wall^41^. The fluorescence signal (Fig. 3A) was low at this point because the liquid film on the inner surface of the flask contains only a small number of fluorescent molecules. In contrast, the transmitted light signal (Fig. 3B) maintained a high constant value because most of the light passes through the liquid film and reaches the detector. The arrival of the leading edge of the rotating bulk liquid in front of the optical fibre or the detector resulted in an abrupt signal change. The fluorescence signal rose sharply until the maximum amount of bulk liquid was within the detection area, corresponding to the increasing number of fluorescent molecules (Fig. 3A). As the viscosity of the fluid increased, the arrival of the bulk liquid was progressively delayed and the rotation was phase-shifted relative to the direction of the centrifugal acceleration. In contrast to the fluorescence signal, the transmitted light signal declined sharply as more light was absorbed by the bulk liquid (Fig. 3B). However, the same response to increasing viscosity was apparent: the higher the viscosity, the later the change in the signal because the arrival of the leading edge of the rotating bulk liquid was progressively delayed. The angle of the leading edge of the bulk liquid is an easily detected characteristic, realized by robust and precise time-lapse measurements. Both the fluorescent and transmitted light signals contain this information, which is all that needs to be extracted to determine the viscosity. The remaining components of the signal (and the absolute values) are not required for the calculation and can be ignored.

**Figure 4:**
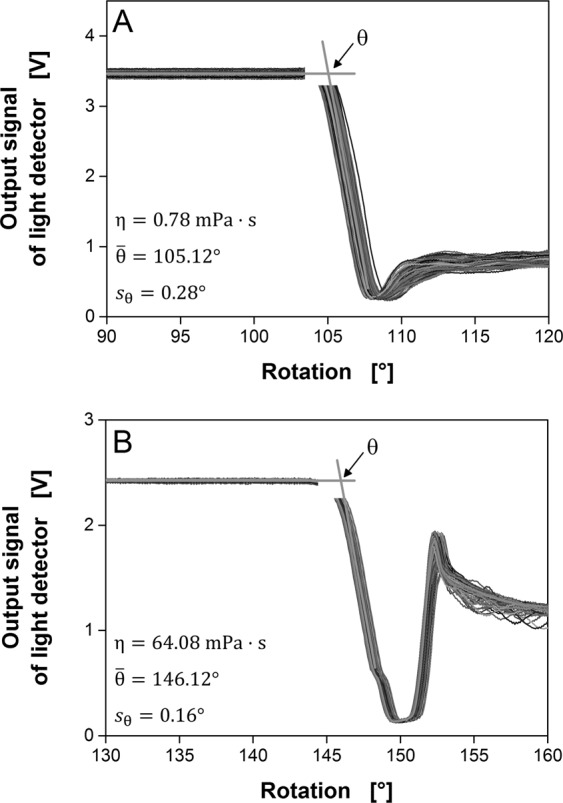
Determination of the angle θ between the zero point and leading edge of the bulk liquid based on the raw signal for 200 rotations (transmitted light measurement). The angle θ is determined by the intersection of two linear fits (light grey lines). The mean $\bar{{\rm{\theta }}}$ and standard deviation s_θ_ are shown. Flask volume = 250 mL, liquid volume = 20 mL, shaking frequency = 350 rpm, shaking diameter = 50 mm, temperature = 30 °C. (**A**) H_2_O, η = 0.78 mPa·s; (**B**) Aqueous 7% (w/w) PVP solution, η = 64.08 mPa·s. Figure adapted from Sieben^19^.

Figure 3 shows representative signals for one rotation, whereas Fig. 4 shows the raw transmitted light signals over 200 rotations (duration: 34.3 s at a shaking frequency of 350 rpm). To illustrate the precision of the measurement and to determine θ, the 200 signals are zoomed to the relevant part, i.e. the arrival of the bulk liquid^19^. The entire signal dataset is provided as Supplementary Information Fig. S1. The leading edge of the rotating bulk liquid was determined by the intersection of two linear fits (light grey lines). The first fit was applied to the data range of the horizontal signal, when there is no bulk liquid in front of the detector, whereas the second fit was applied to the data range during the steep signal decrease. This procedure can be reiterated for as many rotations as desired. The resulting θ angles can be used to calculate the mean $\bar{{\rm{\theta }}}$ and standard deviation s_θ_. For water, $\bar{{\rm{\theta }}}$ = 105.12° with a standard deviation of s_θ_ = 0.28° (Fig. 4A), whereas for an aqueous PVP solution of 64.08 mPa∙s, $\bar{{\rm{\theta }}}$ = 146.12° with a standard deviation of s_θ_ = 0.16° (Fig. 4B) under the given conditions^19^. With increasing viscosity, the signal scatter decreases.

### Calibration of θ and viscosity for Newtonian fluids

**Figure 5:**
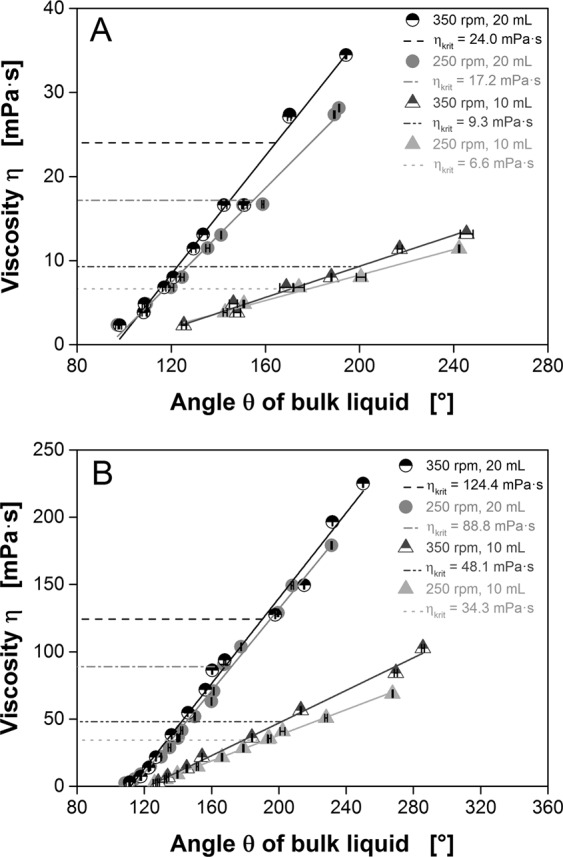
Calibration of the angle θ and the viscosity for varying shaking frequencies and filling volumes. Aqueous liquids with different viscosities were prepared by dissolving different amounts of PVP. Dashed lines represent the critical viscosities η_crit_ according to Büchs *et al*.^36^. At higher viscosities, the out-of-phase phenomenon appears. Black error bars indicate the standard deviation of 20 rotations. Temperature = 30 °C. (**A**) Shaking diameter = 25 mm. **(B**) Shaking diameter = 50 mm.

Having measured the position of a rotating fluid in a shake flask in relation to the direction of centrifugal acceleration, the data must be converted into a viscosity signal^19^. This requires a calibration with model fluids using a conventional rheometer. Fifteen aqueous PVP solutions of different concentrations were chosen as model fluids, and the leading edge of the rotating bulk liquid was measured in a temperature controlled room at either 30 °C or 37 °C. For each data point, 20 rotations were evaluated and averaged, which took 3.4 s at a shaking frequency of 350 rpm, or 4.8 s at 250 rpm. In parallel, the viscosity of the fluids was measured using a conventional rheometer at the same temperatures, and was plotted as a function of the leading edge angle θ of the bulk liquid (Fig. 5). We calculated linear correlations with slopes as a function of shaking diameter, shaking frequency and the filling volume of the shake flask. Although different shaking frequencies caused only minor variations in the calibration functions, varying the filling volume caused significant differences in the slope. At a filling volume of 10 mL, minor changes in viscosity caused a major displacement of the bulk liquid compared to a filling volume of 20 mL. This phenomenon was enhanced at lower shaking diameters. A shaking diameter of 25 mm (Fig. 5A) caused a larger shift in the bulk liquid compared to a shaking diameter of 50 mm (Fig. 5B) under the same conditions^19^. It is therefore possible to adjust the sensitivity of the device by careful selection of the shaking parameters. However, the shaking parameters also have a major impact on the critical viscosity at which the out-of-phase phenomenon occurs (represented by the dotted lines in Fig. 5). Therefore, care must be taken to ensure an in-phase operating range when selecting shaking parameters. Although Fig. 5 also shows out-of-phase operating conditions (above the corresponding dotted lines), the calibration still appears valid. After successful calibration, this novel viscosity measuring method can be applied to fluids of unknown viscosity. As an example, diluted glycerol and sucrose solutions were measured on both, a conventional cone-plate viscometer and on our new viscosity measuring device (shaker viscometer) (Supplementary Information Fig. S3). To account for the increased density of these solutions compared to water, a density factor has been included to calculate their respective viscosity. The Influence of the density on the leading edge angle θ is shown in Supplementary Information Fig. S2. As explained in the last paragraph, a smaller shaking diameter leads to a larger shift in the bulk liquid facilitating a more accurate measurement especially for low viscosity values. Therefore, for viscosity values up to 20 mPa∙s a shaking diameter of 25 mm was used while higher viscosity values were measured at a shaking diameter of 50 mm. A best fit calculation of this data leads to a slope of 1.00 that equals the slope of the equality line (y = x). The y-intercept indicates a small offset of 0.93 mPa∙s, which could be caused by the uneven wall thickness of the used shake flasks and could be avoided by using standardized sample vessels. All in all, the resulting viscosity values obtained with the shaker viscometer are highly comparable to the viscosity values measured on a cone-plate viscometer, demonstrating that the here presented viscosity measuring system works reliably.

### Effective shear rates and non-Newtonian fluids

Unlike Newtonian fluids, non-Newtonian fluids require experiments at different shear rates and the shear rate must be known to determine the apparent viscosity under the relevant operating conditions. In cone-plate rheometers uniform shearing of the sample over the conical radius provides defined shear conditions. However, not all types of rheometers meet this gold standard. In a capillary viscometer the shear rate varies depending on the parabolic flow of the fluid, but can be calculated based on the know flow profile. Mixer-type rheometers as well as the here presented new viscosity measuring device do also not provide uniform shearing of the sample over the whole liquid volume, meaning that the local shear rate varies within the sample. Nevertheless, those devices are essential for the viscosity measurement of fluids containing large dispersed particles where cone-plate or capillary viscometers reach their limits. Hence, a representative effective shear rate must be determined in those systems. In shake flasks, the shear rate depends on the fluid properties (consistency factor *K* and flow behaviour index *m*) and the shaking parameters (shaking frequency, filling volume and flask diameter)^19,46^. *K* and *m* can be determined with a simple two-point measurement using the measuring device described herein (see Supplementary Information). The shaking parameters can in principle be selected freely, but in practice they are constrained by the maximum shaking frequency that can be applied at a fixed shaking diameter without loss of balance and excess vibration. Furthermore, out-of-phase operating conditions should be avoided.

**Figure 6:**
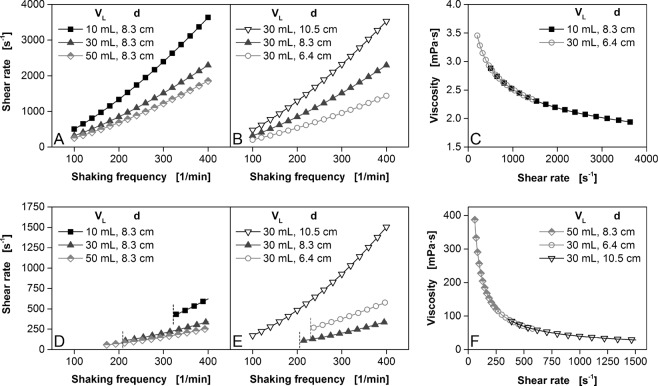
Simulation of shear rates and flow curves as functions of various shaking parameters for two theoretical fluids. V_L_ = filling volume, d = shake flask diameter: d = 6.4 cm corresponds to a nominal shake flask volume of 100 mL, d = 8.3 cm to 250 mL and d = 10.5 cm to 500 mL. Shaking diameter = 70 mm. Liquid density = 1000 kg/m^3^. Equations for the simulation are provided in the Supplementary Information. Data points are exclusively plotted for in-phase shaking conditions. Vertical dotted lines indicate the beginning of in-phase operating conditions. For clarity, only 1 in 10 data points is shown. (**A**–**C**) K = 10 mPa·s^m^, m = 0.8 **(D**–**F**) K = 10,000 mPa·s^m^, m = 0.2. Figure adapted from Sieben^19^.

Figure 6A,B,D,E shows a simulation of the shear rate (calculated using Equations SI1–SI6, Supplementary Information) as a function of various parameters for two theoretical fluids (for further fluids, see Supplementary Information Fig. S4). As is the case for rotary viscometers, the shear rate increases with increasing shaking frequency. In contrast, the shear rate decreases with increasing filling volume (Fig. 6A,D). Further changes in shear rate can be achieved by altering the flask diameter^19^. Although Fig. 6B shows a proportionality between shake flask diameter and shear rate, this trend changes under the conditions illustrated in Fig. 6E. The influence of the shake flask diameter on the shear rate must therefore be considered for each fluid^19^. In the plotted curves in Fig. 6D,E, specific parts at low shaking frequencies are not shown (boundaries indicated by short dotted vertical lines). Due to the higher consistency factor, this fluid has a significantly higher viscosity than the fluid shown in Fig. 6A,B. This results in out-of-phase working conditions for these parts, which should not be considered as suitable measuring conditions. These problems can be avoided by increasing the shaking diameter, because the latter does not influence the shear rate^46^ but nevertheless reduces the probability of out-of-phase conditions^36^. Depending on the range of shear rates used to plot the flow curve, it is sufficient to alter only the shaking frequency because there is a positive correlation between these parameters^19^. For example, for a theoretical fluid where *K* = 10 mPa·s^m^ and *m* = 0.8, shear rates of 500–3600 s^−1^ can be achieved (Fig. 6A,C) at shaking frequencies of 100–400 rpm for a filling volume of 10 mL and a flask diameter of 8.3 cm (Fig. 6A,C). Additional shear rate ranges can be covered by changing the flask diameter and/or filling volume (Fig. 6C,F)^19^. For the above-mentioned fluid, increasing the filling volume to 30 mL and reducing the flask diameter to 6.4 cm generates sheer rates in the range 200–1400 s^−1^ (Fig. 6C) at shaking frequencies of 100–400 rpm. Compared to the above range, lower shear rates of 200–500 s^−1^ can also be generated under these conditions. For shear rates of 500–1400 s^−1^, the conditions overlap (Fig. 6C). Accordingly, Fig. 6F shows the overlapping flow curves at various shaking parameters for a theoretical fluid where *K* = 10,000 mPa·s^m^ and *m* = 0.2. Our new device allows multiple sample vessels to be analysed in parallel, so different fluids can be tested in parallel or several filling volumes and vessel diameters can be used to achieve different shear rates^19^. Figure S5 substantiates the simulated flow curve data presented in Figs 6 and S4 with an exemplary measurement of a 0.5% (w/w) aqueous alginate solution using both, a cone-plate viscometer and our shaker viscometer. The comparison of both flow curves illustrates that the apparent viscosities obtained by the shaker viscometer are very close to the ones measured on the cone-plate viscometer. This also implies that the effective shear rates in shake flasks calculated by the correlation of Giese *et al*.^46^ is applicable.

## Discussion

We have developed a contact-free method to measure viscosity based on the analysis of liquid distribution in an orbitally shaken sample vessel. As the viscosity increases, the rotation of the liquid is phase-shifted relative to the direction of centrifugal acceleration. Two different optical methods to determine the position of the liquid in transparent vessels are described herein. An additionally developed method using radar allows the measurement in opaque vessels. Using model fluids, we established a correlation between viscosity and the position of the leading edge of the rotating bulk liquid for liquids with a density similar to or higher than water. In this setup, the shear rate is dependent on the shaking parameters as well as the fluid characteristics. This establishes the operational range of the method and the protocol used to determine flow curves.

The new contact-free viscometer uses inexpensive components and disposable sample vessels, enabling the parallel measurement of multiple samples. This allows a high-throughput sample analysis while eliminating costs associated with cleaning, and the robust design ensures durability. The viscosity-dependent signal is periodic in nature, so accuracy is increased by averaging over multiple readings. Our device is also much more suitable than traditional concentric cylinders and plate-plate rheometers for the analysis of fluids with large dispersed particles because there are no restrictions caused by gap sizes, and it can handle smaller sample volumes than mixer-type rheometers. The working principle of this novel viscometer is applicable as an online measuring technique to monitor viscosity changes during chemical or biological reactions. Further fields of application include quality assurance and quality control for many parallel samples, as well as the routine consistency testing of viscous products. The contact-free nature of the device makes it particularly suitable for the analysis of toxic and infectious samples, and with closed sample vessels it is also suitable for the analysis of volatile samples, which are difficult to measure using conventional rotation and oscillation rheometers due to rapid evaporation.

## Materials and Methods

### Preparation of fluorescein solution and Oregon Green® 488

Fluorescein sodium salt (F6377-1006, Sigma Aldrich Co. LLC., St. Louis, MO, USA, *λ*_*ex*_ = 470 nm, *λ*_*em*_ = 524 nm) was prepared as a 2.5 mM stock solution in 0.1 M phosphate buffer at pH 8.0 because its optical properties are pH dependent^47^. Test liquids were supplemented with fluorescein to a final concentration of 2.5 µM immediately before measurement.

Oregon Green® 488 (D6145, Molecular Probes, Inc., Eugene, OR, USA) is a fluorinated analog of fluorescein exhibiting the same excitation and emission wavelength and was used as an alternative fluorescent dye. According to the manufacturer’s instruction, 10 mg of the dye was dissolved in 1 mL dimethyl sulfoxide. This stock solution was stored protected from light at room temperature for one week at most. Liquids to be examined were supplemented with Oregon Green® 488 to a final concentration of 2.5 µM directly before measurement^19^.

### Image capture

Images of liquid movement in shake flasks were captured using a rotating camera as previously described^48,49^. We used a GoPro HERO4 black action camera (GoPro, San Mateo, CA, USA)^19^.

### Viscous model liquids and rheometer measurements

Aqueous PVP (Luviskol K90, BASF, Ludwigshafen, Germany) solutions were prepared from a 15% (w/w) stock solution in 0.1 M phosphate buffer (pH 8.0) for fluorescence measurement or a 15% (w/w) stock solution in distilled water for transmitted light measurement. The solvents were preheated to 60 °C before adding PVP powder, and agitated on a magnetic stirrer for 2 days at 60 °C until the powder had completely dissolved. Dilution series were prepared in 0.1 M phosphate buffer (pH 8.0) or distilled water as appropriate, and were stored at 30 °C or 37 °C, respectively^19^. Dilution series of pure glycerol (VWR International, Radnor, PA USA) and sucrose (Carl Roth GmbH, Karlsruhe, Germany) as well as the 0.5% (w/w) alginate solution (Alginate sodium salt, Carl Roth GmbH, Karlsruhe, Germany) were prepared with distilled water and stored at 30 °C. Viscous flow behaviour was analysed before each experiment using a MCR 301 rheometer (Anton Paar, Stuttgart, Germany) equipped with a cone (CP50-0.5/TG, cone truncation 54 µM, cone angle 0.467°) within the shear range 10–5000 s^−1^ at 37 °C or 30 °C, as appropriate. Aqueous PVP solutions show slight shear thinning characteristics at increasing concentrations (Supplementary Information Fig. S6), so the effective shear rates and resulting apparent viscosity values were determined as previously described^19,46^. To determine the viscosity of sample fluids with a density different from the reference PVP solutions the sample viscosity is converted using the density quotients (η_sample_ = η_reference_ ∙ ρ_sample_/ρ_reference_). The density viscosity relation was validated based on measurements of fluids with a defined density between 1.00 and 2.44 g/mL (Supplementary Data).

### Sodium polytungstate – sucrose solutions

Sodium polytungstate (SPT, Na6[H2W12O40]) (SPT-2, TC-Tungsten Compounds GmbH, Grub am Forst, Germany) stock solutions of different concentrations (40%, 50%, 60%, 70%, 75%, 80% (w/w)) (Tables S4–S11) were prepared by dissolving the granulate in 0.1 M phosphate buffer pH 8.0. The desired amount of sucrose was added to the preheated stock solution which was mixed on a magnetic stirrer with heater at 60 °C until the sugar was completely dissolved. Dilution series were prepared with 0.1 M phosphate buffer pH 8.0 and stored at 37 °C. Viscous flow behavior of the solutions was analyzed prior to each experiment in a cone-plate rheometer. Effective shear rates and resulting apparent viscosity values were determined according to Giese *et al*.^19,46^.

## Supplementary information

Supplementary Information

## Data Availability

The datasets generated and/or analysed during the current study are available from the corresponding author by request.
