# Supplementary material for: Contact-free determination of viscosity in multiple parallel samples
Source: Sci Rep. 2019 Jun 6;9:8335. doi: 10.1038/s41598-019-44859-z (PMC6554296; doi:10.1038/s41598-019-44859-z)
Supplement: Supplementary file 1 — Supplementary Information [file 41598_2019_44859_MOESM1_ESM.docx]

**Supplementary Information of the Research Article**

**“Contact-free determination of viscosity in multiple parallel samples”**

Michaela Sieben^1^

Rene Hanke^1^

Jochen Büchs^1^

^1^RWTH Aachen University, AVT – Biochemical Engineering, Aachen, Germany

**Correspondence:** Prof. Dr.-Ing. Jochen Büchs, RWTH Aachen University, AVT – Biochemical Engineering, Forckenbeckstraße 51, D-52074 Aachen, Germany.

**E-mail**: Jochen.Buechs@avt.rwth-aachen.de





**Supplementary Figure S1: Raw signal for the transmitted light measurement over 200 rotations**. Flask volume = 250 mL, liquid volume = 20 mL, shaking frequency = 350 rpm, shaking diameter = 50 mm, temperature = 30°C. **A** H_2_O with η = 0.78 mPa·s; **B** Aqueous 7% (w/w) PVP solution with η = 64.08 mPa·s. Figure adapted from Sieben^19^.

**Influence of the density on the leading edge angle θ at various viscosities**

To investigate the influence of the density on the liquid distribution in shake flasks at various viscosities, model fluids differing in density and viscosity were required. Aqueous PVP solutions had already been used to investigate the flow behavior of viscous fluids in shake flasks^36,46^ and were therefore a good first choice^19^. Their density is about 1.0 g/mL. Glycerol is a well-known highly viscous compound with a density of about 1.26 g/mL at 37°C meaning that it is denser than water. Sodium polytungstate (SPT) (Na6[H2W12O40]) was used as a heavy liquid with a density larger than 1.26 g/mL. It is non-toxic and non-flammable and used for gravity separation (sink and swim analysis) and density gradient centrifugation^50–52^. Depending on the concentration, it reaches a density of up to 3.1 g/mL. Sucrose was added as a viscosity modifying agent. As a result, highly viscous fluids of high density could be established. In order to have as many different combinations of density and viscosity as possible, differently concentrated SPT stock solutions were prepared and a different amount of sucrose was dissolved in each of them. Then, dilution series were prepared from these initial solutions. Table S4 - Table S11 give an overview of all model fluids investigated with their concentration, viscosity, density and pH-value. The viscosity of these model fluids was determined with a conventional cone-plate rheometer^19^. The experiments were performed using the fluorescence measuring set-up with 2.5 µM Oregon Green® 488 as a fluorescent dye.

In Figure S2 A the dynamic viscosity was plotted as a function of the leading edge angle θ of the bulk liquid. This demonstrates that the density has a considerable influence on the leading edge angle θ for fluids of different density but equal viscosity. This finding is reasonable as a change in density leads to a change in mass at a constant filling volume of the shake flask. As a result, the centrifugal force increases with increasing density. Due to the higher centrifugal force, heavy fluids show a smaller shift of the bulk liquid compared to light fluids at the same viscosity. If the kinematic viscosity (ν=η/ρ) is plotted instead of the dynamic viscosity as function of the leading edge angle θ of the bulk liquid, the data points perfectly match in the low viscosity range and only diverge a little in the high viscosity range (Figure S4) ^19^. This might be due to pipetting errors of the highly viscous liquids.


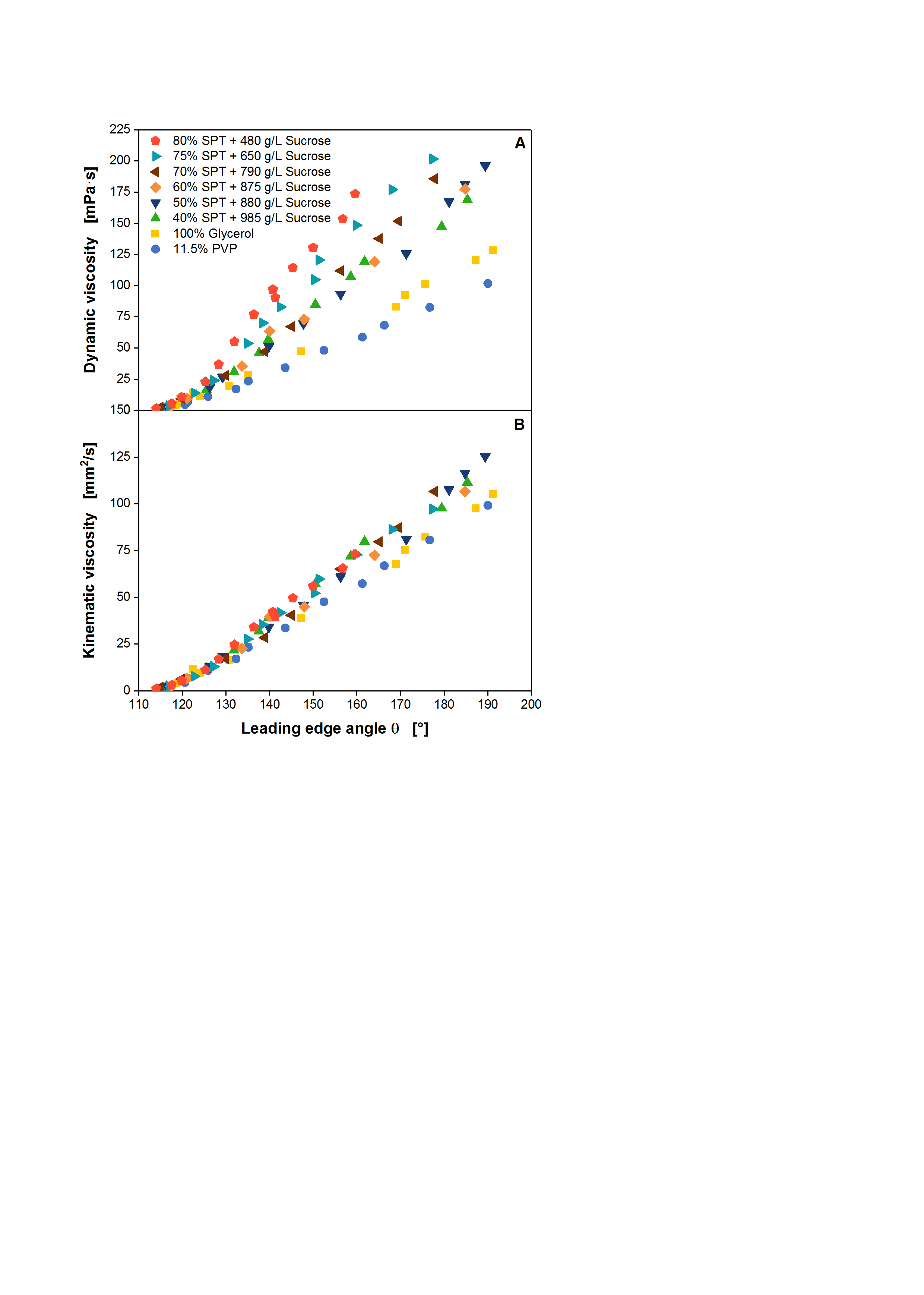


**Supplementary Figure S2: Influence of the density on the leading edge angle θ of the bulk liquid**

Fluorescence measuring set-up, Oregon Green® 2.5 µM, flask volume = 250 mL, liquid volume = 20 mL, shaking frequency = 350 rpm, shaking diameter = 50 mm, temperature = 37°C, for model fluids weight percent and density of dilution series are specified in Table S4 - Table S11. **A** Dynamic viscosity as function of the angle θ. **B** Kinematic Viscosity as function of the angle θ. Figure adapted from Sieben^19^.

**

**

**Supplementary Figure S3: Parity plot comparing the viscosity measurement of aqueous glycerol and sucrose solutions on a conventional cone-plate viscometer and the here described new shaker viscometer**

Flask volume = 250 mL, liquid volume = 20 mL, shaking frequency = 350 rpm, shaking diameter = 25 mm (open symbols) and 50 mm (filled symbols), temperature = 30°C, aqueous glycerol and sucrose solutions of different concentrations^53,54^ . Black error bars indicate the standard deviation of triplicates.





**Supplementary Figure S4: Simulation of shear rates and flow curves as a function of various shaking parameters for two additional theoretical fluids.** V_L_ = filling volume, d = shake flask diameter: d = 6.4 cm corresponds to a nominal shake flask volume of 100 mL, d = 8.3 cm to 250 mL and d = 10.5 cm to 500 mL. Shaking diameter = 70 mm. Liquid density = 1000 kg/m^3^. Equations for the simulation are given in the extended data. Data points are exclusively plotted for in-phase shaking conditions. Vertical dotted lines indicate the beginning of in-phase operating conditions. For clarity, only 1 in 10 data points is shown. **A-C** *K* = 100 mPa·s^m^, *m* = 0.8 **D-F** *K* = 1,000 mPa·s^m^, *m* = 0.2. Figure reused from Sieben^19^.

**

**

**Supplementary Figure S5: Flow curve of a 0.5% (w/w) aqueous alginate solution comparing a cone-plate viscometer and the here described shaker viscometer.**

Flask volume = 250 mL, shaking diameter = 50 mm, temperature = 30°C, different shear rates were set by varying the shaking frequency from 150 to 350 rpm and using liquid volumes of 10, 20 and 30 mL.

**Supplementary Data Table S1: Viscosity of aqueous PVP solutions with different concentrations at 25°C**

Flask volume = 250 mL, liquid volume = 30 mL, shaking frequency = 150 rpm, shaking diameter = 50 mm, temperature = 25°C. Because PVP is a slightly shear-thinning fluid, its viscosity depends on the shear rate which is influenced by the shaking parameters.

| PVP concentration [%(w/w)] | Apparent viscosity  at 25°C [mPa·s] |
| --- | --- |
| 1 | 2.3 |
| 5 | 16 |
| 7 | 34.6 |
| 8.5 | 53.6 |
| 9.5 | 85.5 |
| 10.5 | 114.7 |

**Supplementary Data Table S2: Viscosity of aqueous PVP solutions with different concentrations at 37°C**

Flask volume = 250 mL, liquid volume = 20 mL, shaking frequency = 350 rpm, shaking diameter = 50 mm, temperature = 37°C. Because PVP is a shear-thinning fluid, its viscosity depends on the shear rate which is influenced by the shaking parameters.

| PVP concentration [%(w/w)] | Apparent viscosity  at 37°C [mPa·s] |
| --- | --- |
| 2 | 4.0 |
| 5 | 18.9 |
| 6 | 34.9 |
| 8 | 69.1 |

**Supplementary Data Table S3: Viscosity of aqueous PVP solutions with different concentrations at 30°C**

Flask volume = 250 mL, liquid volume = 20 mL, shaking frequency = 350 rpm, shaking diameter = 50 mm, temperature = 30°C. Because PVP is a shear-thinning fluid, its viscosity depends on the shear rate which is influenced by the shaking parameters.

| PVP concentration [%(w/w)] | Apparent viscosity  at 30°C [mPa·s] |
| --- | --- |
| 1 | 3.0 |
| 5 | 38.1 |
| 7 | 71.8 |
| 8.5 | 127.5 |





**Supplementary Figure S6: Shear thinning behaviour of aqueous PVP solutions with different concentrations (% (w/w)).** As the concentration and viscosity increases, the solution behaves less like a Newtonian fluid. T = 30°C. Figure reused from Sieben^19^.

**Supplementary Data Table S4: Dilution series of glycerol stock solution**

Flask volume = 250 mL, liquid volume = 20 mL, shaking frequency = 350 rpm, shaking diameter = 50 mm, temperature = 37°C. Table reused from Sieben^19^.

| Concentration  [% (w/w)] | Viscosity  [mPa·s] | Density  [g/mL] | pH  [-] |
| --- | --- | --- | --- |
| 100 | 372.2 | 1.24 | - |
| 99 | 270.6 | 1.25 | 8.13 |
| 98 | 240.8 | 1.24 | 8.34 |
| 97 | 209.6 | 1.23 | 8.27 |
| 96 | 174.5 | 1.24 | 8.47 |
| 95 | 150.6 | 1.23 | 8.47 |
| 94 | 137.5 | 1.23 | 8.49 |
| 93 | 122.7 | 1.23 | 8.5 |
| 92 | 125.5 | 1.24 | 8.47 |
| 91 | 100.2 | 1.23 | 8.63 |
| 90 | 87.8 | 1.22 | 8.57 |
| 85 | 51.9 | 1.21 | 8.52 |
| 80 | 29.6 | 1.19 | 8.48 |
| 75 | 24.4 | 1.19 | 8.45 |
| 70 | 16.2 | 1.18 | 8.4 |
| 65 | 12.3 | 1.16 | 8.38 |
| 60 | 7.8 | 1.15 | 8.33 |
| 55 | 6 | 1.14 | 8.28 |
| 50 | 4.6 | 1.12 | 8.24 |

**Supplementary Data Table S5: Dilution series of PVP stock solution**

Flask volume = 250 mL, liquid volume = 20 mL, shaking frequency = 350 rpm, shaking diameter = 50 mm, temperature = 37°C. Table reused from Sieben^19^.

| Concentration [% (w/w)] | Viscosity [mPa·s] | Density  [g/mL] | pH  [-] |
| --- | --- | --- | --- |
| 11.5 | 208.2 | 1.03 | 7.8 |
| 11 | 177.8 | 1.03 | 7.8 |
| 10.5 | 169.7 | 1.03 | 7.8 |
| 10 | 117.9 | 1.02 | 7.78 |
| 9.5 | 100.7 | 1.02 | 7.8 |
| 9 | 89.5 | 1.02 | 7.8 |
| 8.5 | 70.9 | 1.01 | 7.81 |
| 8 | 60.0. | 1.02 | 7.83 |
| 7 | 44.6 | 1.01 | 7.87 |
| 6 | 27.2 | 1.01 | 7.89 |
| 5 | 21.9 | 1.01 | 7.91 |
| 4 | 9.8 | 1.01 | 7.96 |
| 3 | 5.4 | 1 | 8 |
| 2 | 5 | 1 | 8.02 |
| 1 | 2.5 | 1 | 8.05 |

**Supplementary Data Table S6: Dilution series of 40% (w/w) SPT stock solution with 895 g/L sucrose**

Flask volume = 250 mL, liquid volume = 20 mL, shaking frequency = 350 rpm, shaking diameter = 50 mm, temperature = 37°C. Table reused from Sieben^19^.

| Sucrose concentration  [% (w/w)] | Viscosity  [mPa·s] | Density  [g/mL] | pH  [-] |
| --- | --- | --- | --- |
| 895 | 251.2 | 1.53 | 6.79 |
| 890 | 235.2 | 1.52 | 6.80 |
| 880 | 202.5 | 1.51 | 6.82 |
| 870 | 180.3 | 1.57 | 6.79 |
| 860 | 148.2 | 1.50 | 6.79 |
| 845 | 115.5 | 1.50 | 6.80 |
| 830 | 101.3 | 1.49 | 6.80 |
| 815 | 77.3 | 1.48 | 6.80 |
| 780 | 56.8 | 1.45 | 6.78 |
| 760 | 43.9 | 1.24 | 6.80 |
| 725 | 29.6 | 1.42 | 6.80 |
| 650 | 14.9 | 1.37 | 6.81 |
| 350 | 2.6 | 1.20 | 6.93 |

**Supplementary Data Table S7: Dilution series of 50% (w/w) SPT stock solution with 880 g/L sucrose**

Flask volume = 250 mL, liquid volume = 20 mL, shaking frequency = 350 rpm, shaking diameter = 50 mm, temperature = 37°C. Table reused from Sieben^19^.

| Sucrose concentration  [% (w/w)] | Viscosity  [mPa·s] | Density  [g/mL] | pH  [-] |
| --- | --- | --- | --- |
| 880 | 260.1 | 1.60 | 6.80 |
| 875 | 279.8 | 1.59 | 5.46 |
| 865 | 202.9 | 1.58 | 5.58 |
| 860 | 210.5 | 1.58 | 6.79 |
| 855 | 210.8 | 1.58 | 5.65 |
| 850 | 187.5 | 1.57 | 5.70 |
| 840 | 146.2 | 1.56 | 6.80 |
| 820 | 112.5 | 1.56 | 6.80 |
| 800 | 83.8 | 1.54 | 6.80 |
| 780 | 66.5 | 1.53 | 6.80 |
| 750 | 50.4 | 1.50 | 6.12 |
| 690 | 26.8 | 1.45 | 6.28 |
| 650 | 16.6 | 1.44 | 6.81 |
| 350 | 2.6 | 1.23 | 6.90 |

**Supplementary Data Table S8: Dilution series of 60% (w/w) SPT stock solution with 875 g/L sucrose**

Flask volume = 250 mL, liquid volume = 20 mL, shaking frequency = 350 rpm, shaking diameter = 50 mm, temperature = 37°C. Table reused from Sieben^19^.

| Sucrose concentration  [% (w/w)] | Viscosity  [mPa·s] | Density  [g/mL] | pH  [-] |
| --- | --- | --- | --- |
| 875 | 98.7 | 1.71 | 5.26 |
| 860 | 83.4 | 1.70 | 5.43 |
| 830 | 64.6 | 1.68 | 5.67 |
| 800 | 53.4 | 1.66 | 5.35 |
| 775 | 75.8 | 1.61 | 5.08 |
| 750 | 30.0 | 1.63 | 5.65 |
| 700 | 19.2 | 1.57 | 5.80 |
| 550 | 6.0 | 1.43 | 5.80 |
| 350 | 2.3 | 1.28 | 5.90 |

**Supplementary Data Table S9: Dilution series of 70% (w/w) SPT stock solution with 790 g/L sucrose**

Flask volume = 250 mL, liquid volume = 20 mL, shaking frequency = 350 rpm, shaking diameter = 50 mm, temperature = 37°C. Table reused from Sieben^19^.

| Sucrose concentration  [% (w/w)] | Viscosity  [mPa·s] | Density  [g/mL] | pH  [-] |
| --- | --- | --- | --- |
| 790 | 188.5 | 1.81 | 6.55 |
| 785 | 162.8 | 1.80 | 6.57 |
| 780 | 163.9 | 1.79 | 6.57 |
| 775 | 140.4 | 1.78 | 6.63 |
| 770 | 131.0 | 1.78 | 6.67 |
| 765 | 118.9 | 1.77 | 6.57 |
| 760 | 116.2 | 1.76 | 6.57 |
| 755 | 100.3 | 1.75 | 6.57 |
| 750 | 96.6 | 1.74 | 6.57 |
| 725 | 68.5 | 1.72 | 6.60 |
| 700 | 50.9 | 1.70 | 6.61 |
| 650 | 27.7 | 1.64 | 6.81 |
| 550 | 10.6 | 1.52 | 6.67 |

**Supplementary Data Table S10: Dilution series of 75% (w/w) SPT stock solution with 650 g/L sucrose**

Flask volume = 250 mL, liquid volume = 20 mL, shaking frequency = 350 rpm, shaking diameter = 50 mm, temperature = 37°C. Table reused from Sieben^19^.

| Sucrose concentration  [% (w/w)] | Viscosity  [mPa·s] | Density  [g/mL] | pH  [-] |
| --- | --- | --- | --- |
| 650 | 432.6 | 2.16 | 4.99 |
| 640 | 337.3 | 2.15 | 5.35 |
| 630 | 253.6 | 2.11 | 5.53 |
| 625 | 212.8 | 2.12 | 5.43 |
| 620 | 235.7 | 2.11 | 6.05 |
| 610 | 176.1 | 2.07 | 6.57 |
| 600 | 145.0 | 2.07 | 6.35 |
| 590 | 107.5 | 2.03 | 6.34 |
| 580 | 90.3 | 2.02 | 6.43 |
| 570 | 73.6 | 2.00 | 6.42 |
| 550 | 50.1 | 1.96 | 6.46 |
| 500 | 27.6 | 1.86 | 6.50 |
| 450 | 12.9 | 1.78 | 6.77 |
| 300 | 3.5 | 1.51 | 6.87 |

**Supplementary Data Table S11: Dilution series of 80% (w/w) SPT stock solution with 480 g/L sucrose**

Flask volume = 250 mL, liquid volume = 20 mL, shaking frequency = 350 rpm, shaking diameter = 50 mm, temperature = 37°C Table reused from Sieben^19^.

| Sucrose concentration  [% (w/w)] | Viscosity  [mPa·s] | Density  [g/mL] | pH  [-] |
| --- | --- | --- | --- |
| 480 | 300.6 | 2.44 | 5.54 |
| 460 | 173.4 | 2.37 | 5.28 |
| 455 | 156.3 | 2.37 | 4.98 |
| 450 | 133.1 | 2.34 | 5.02 |
| 445 | 114.1 | 2.33 | 5.66 |
| 440 | 99.1 | 2.33 | 5.56 |
| 430 | 85.0 | 2.30 | 5.96 |
| 420 | 64.5 | 2.27 | 6.61 |
| 400 | 40.6 | 2.20 | 6.58 |
| 375 | 24.6 | 2.11 | 6.55 |
| 325 | 10.6 | 1.94 | 6.71 |
| 275 | 5.5 | 1.80 | 6.70 |
| 100 | 1.5 | 1.23 | 6.94 |

**Equations used for the simulation of shear rates and flow curves as a function of various shaking parameters**

The flow behaviour of shear-thinning fluids can be described by the Ostwald-de-Wale law^55^:

| $\eta_{app}=K\cdot{\dot{\gamma}_{eff}}^{(m-1)}$ | (SI1) |
| --- | --- |

.

Büchs et al.^36,56^ investigated the power input into shake flasks (equation SI2) and established an empirical equation that correlates the modified Newton number (equation SI3) and the Reynold’s number (equation SI4).

| ${Ne}^{'}=70{Re}^{-1}+25{Re}^{-0.6}+1.5{Re}^{-0.2}$ | (SI2) |
| --- | --- |

| ${Ne}^{'}=\frac{P}{\rho n^{3}d^{4}V^{\frac{1}{3}}}$ | (SI3) |
| --- | --- |

| $Re=\frac{\rho nd^{2}}{\eta_{app}}$ | (SI4) |
| --- | --- |

Inserting equation SI 1 into equation SI 4 yields:

| $Re=\frac{\rho nd^{2}}{K\cdot{\dot{\gamma}_{eff}}^{(m-1)}}$ | (SI5) |
| --- | --- |

Giese et al.^46^ presented an equation to calculate the effective shear rate in shake flasks as a function of the shaking parameters.

| $\dot{\gamma}_{eff}={2.06}^{\frac{1}{m+1}} {\left( \frac{{V_{L}}^{1/3}}{d} \right)^{\frac{-0.331}{m+1}}\left( \frac{P}{V_{L}}\cdot\frac{1}{K} \right)}^{\frac{1}{m+1}}$ | (SI6) |
| --- | --- |

By combining equations SI2, SI3, SI5 and SI6, shear rates and flow curves can be simulated for various shaking parameters.

**Determination of consistency factor *K* and flow behaviour index *m***

*K* and *m* can be determined with a simple two-point measurement using the measuring device described herein. The apparent viscosity of the liquid sample is determined at either two different shaking frequencies or with two different filling volumes. By knowing the apparent viscosities and applying the Ostwald-de-Waele law (equation SI1) as well as the shear rate equation (equation SI6) for both selected shaking conditions, the consistency factor *K* and the flow behaviour index *m* can be calculated.

$d$ maximal shake flask diameter [m]

$K$ consistency factor [Pa·s^m^]

$m$ flow behavior index [-]

$n$ shaking frequency [s^-1^]

${Ne}^{'}$ modified Newton number [-]

$P$ power input [kW]

$Re$ Reynold’s number [-]

$V_{L}$ liquid volume [m^3^]

$\dot{\gamma}_{eff}$ effective shear rate [s^-1^]

$\eta_{app}$ dynamic apparent viscosity [Pa·s]

$\rho$ liquid density [kg/m^3^]

MATLAB® algorithm for the evaluation of the angle θ based on the transmitted light measurement

clear

clc

M = dlmread('rawdata.txt');

Grad = M(:,1);

dm = size(M);

zm = dm(2);

Winkel = zeros(zm-2,1);

for iw=1:zm-2;

Signal = M(:,iw+2);

for f=1:length(Signal)

Signalnorm(f,1)=(Signal(f,1)-min(Signal))/(max(Signal)-min(Signal));

end

lg_alle = find(Grad>-120.0);

lg = lg_alle(1,1);

values_360 = length(Signal);

values_5 = values_360/72;

d_int = floor(values_5);

gw = 0.002;

B = 1;

m = 0;

n = 1;

while B==true

m=m+1;

n=n+1;

E = trapz(Grad(lg:lg+d_int),Signalnorm(lg:lg+d_int));

F = trapz(Grad(lg+m*d_int:lg+n*d_int),Signalnorm(lg+m*d_int:lg+n*d_int));

if abs(1-F/E)>=gw

B = 0;

end

GradLHF_rg = Grad(lg+m*d_int);

SignalLHF_rg = Signalnorm(lg+m*d_int);

end

koeff_LHF = polyfit(Grad(lg:lg+m*d_int), Signalnorm(lg:lg+m*d_int),1);

hw1 = 0.8;

sw1 = hw1*mean(Signalnorm(lg:lg+m*d_int));

rg_alle = find(Signalnorm(lg:length(Signalnorm))<sw1);

hw2 = 0.90;

hw3 = 0.95;

if isempty(rg_alle)

sw2 = hw2*mean(Signalnorm(lg:lg+m*d_int));

rg_alle = find(Signalnorm(lg:length(Signalnorm))<sw2);

if isempty(rg_alle)

sw3 = hw3*mean(Signalnorm(lg:lg+m*d_int));

rg_alle = find(Signalnorm(lg:length(Signalnorm))<sw3);

if isempty(rg_alle)

display('Fehler: Sichelbeginn nicht detektierbar'); display(iw+2);

else

rg = rg_alle(1,1)+lg;

end

else

rg = rg_alle(1,1)+lg;

end

else

rg = rg_alle(1,1)+lg;

GradAusw = Grad(lg:rg);

SignalnormAusw = Signalnorm(lg:rg);

[slope0]=polyfit(Grad(rg-1:rg+1),Signalnorm(rg-1:rg+1),1);

[slope1]=polyfit(Grad(rg-2:rg+1),Signalnorm(rg-2:rg+1),1);

Steigung1(1,1)=slope0(1,1);

Steigung1(2,1)=slope1(1,1);

i=2;

k=3;

while abs((Steigung1(i,1)-Steigung1(1,1))/Steigung1(1,1))<=0.07

[slope]=polyfit(Grad(rg-k:rg+1),Signalnorm(rg-k:rg+1),1);

i=i+1;

Steigung1(i,1) = slope(1,1);

k=k+1;

end

[slope0_2]=polyfit(Grad(rg-1:rg+1),Signalnorm(rg-1:rg+1),1);

[slope0_1]=polyfit(Grad(rg-1:rg+2),Signalnorm(rg-1:rg+2),1);

Steigung(1,1)=slope0_2(1,1);

Steigung(2,1)=slope0_1(1,1);

ii=2;

kk=3;

while abs((Steigung(ii,1)-Steigung(1,1))/Steigung(1,1))<=0.07

[slope]=polyfit(Grad(rg-1:rg+kk),Signalnorm(rg-1:rg+kk),1);

ii=ii+1;

Steigung(ii,1)=slope(1,1);

kk=kk+1;

end

[slope]=polyfit(Grad(rg-k:rg+kk),Signalnorm(rg-k:rg+kk),1);

Winkel(iw,1)=(slope(1,2)-koeff_LHF(1,2))/(koeff_LHF(1,1)-slope(1,1));

end

end
